# Validating the Amyloid Cascade Through the Revised Criteria of Alzheimer's Association Workgroup 2024 for Alzheimer Disease

**DOI:** 10.1212/WNL.0000000000213675

**Published:** 2025-05-13

**Authors:** Augusto J. Mendes, Federica Ribaldi, Michela Pievani, Cecilia Boccalini, Valentina Garibotto, Giovanni B. Frisoni, for the Alzheimer's Disease Neuroimaging Initiative

**Affiliations:** 1 Laboratory of Neuroimaging of Aging (LANVIE), University of Geneva, Switzerland;; 2 Geneva Memory Center, Department of Rehabilitation and Geriatrics, Geneva University Hospitals, Switzerland;; 3 Laboratory of Alzheimer's Neuroimaging and Epidemiology (LANE), IRCCS Istituto Centro San Giovanni di Dio Fatebenefratelli, Brescia, Italy;; 4 Laboratory of Neuroimaging and Innovative Molecular Tracers (NIMTlab), Geneva University Neurocenter and Faculty of Medicine, University of Geneva, Switzerland;; 5 Division of Nuclear Medicine and Molecular Imaging, Geneva University Hospitals, Switzerland; and; 6 CIBM Center for Biomedical Imaging, Geneva, Switzerland.

## Abstract

**Background and Objectives:**

The amyloid cascade hypothesis posits that Alzheimer disease (AD) progresses from amyloid deposition to tau deposition, neurodegeneration, and eventually cognitive impairment and is the foundation of the revised criteria of Alzheimer's Association Workgroup 2024 (AA-2024). To account for copathologies and cognitive resilience that affect the penetrance of the AD cascade, AA-2024 introduced a 2-dimensional biological-clinical staging framework. We aimed to estimate the proportion of persons along the AD continuum whose biological and clinical trajectories align with the amyloid cascade.

**Methods:**

Cross-sectional data of the Alzheimer's Disease Neuroimaging Initiative (ADNI) cohort were tested in the 4 × 4 biological/clinical staging matrix adapted from the AA-2024 criteria. Biological stages were defined by amyloid and tau-PET burden: stage A (amyloid positivity, A+), stage B (medial temporal tau, A+/T_2MTL_+), stage C (moderate neocortical tau, A+/T_2MOD_+), and stage D (high neocortical tau, A+/T_2HIGH_+). Clinical stages were cognitively unimpaired (stage 1), subtle cognitive impairment (stage 2), mild cognitive impairment (stage 3), and dementia (stages 4–6). Tau-PET cutoffs were defined through the implementation of 5 distinct methods. Participants were categorized into (1) compliant with the amyloid cascade (matrix diagonal), (2) resilient (advanced biological stage—early clinical stage), and (3) copathologic (early biological stage—advanced clinical stage). Observed distributions were compared with hypothetical scenarios with zero and high amyloid cascade penetrance using the χ^2^ test, and differences among the 5 methods were tested using the Cochran Q test.

**Results:**

Two-hundred and fifty-six amyloid-positive individuals (mean age: 72.7 years; 51% female) from the ADNI cohort were considered. The proportion of participants compliant with the amyloid cascade was between 31% (95% CI 25%–37%) and 36% (95% CI 30%–42%) depending on the tau-PET cutoff method. The observed number of individuals compliant with the amyloid cascade was higher than in the zero-penetrance scenario but lower than in the high-penetrance distribution (*p* < 0.01). The proportion of copathologic (17%–63%) and resilient (6%–52%) individuals varied widely by tau-PET cutoff (*p* < 0.001).

**Discussion:**

Only approximately one-third of persons with an AA-2024 diagnosis of AD complied with the predictions of the amyloid cascade hypothesis. These results suggest the heterogeneity in how clinical symptoms and pathology are coupled along the AD continuum, which has significant implications for interpreting completed antiamyloid clinical trials and designing future studies.

## Introduction

The Alzheimer's Association Workgroup revised the criteria for diagnosis and staging of Alzheimer disease (AD) in 2024 (AA-2024).^1^ These criteria have been proposed to drive the inclusion of patients in clinical trials of drugs targeting disease biology. They operationalize the progression of AD along a 2-dimensional clinical-biological trajectory where patients enter the AD trajectory with isolated amyloid deposition (biological stage A) when still cognitively intact (clinical stage 1). They develop tau deposits in the medial temporal lobe (biological stage B) and develop subtle cognitive impairment (clinical stage 2). Later on, tau deposition extends to the neocortex and cognitive impairment sets in, initially with moderate neocortical tau burden (biological stage C) and mild cognitive impairment (clinical stage 3) and finally with severe neocortical tau burden (biological stage D) and dementia (mild, moderate, and severe corresponding to clinical stages 4–6).^2^ Neurodegeneration is closely related in time and topography to tau deposition and is not featured in the model.

**Figure 1:**
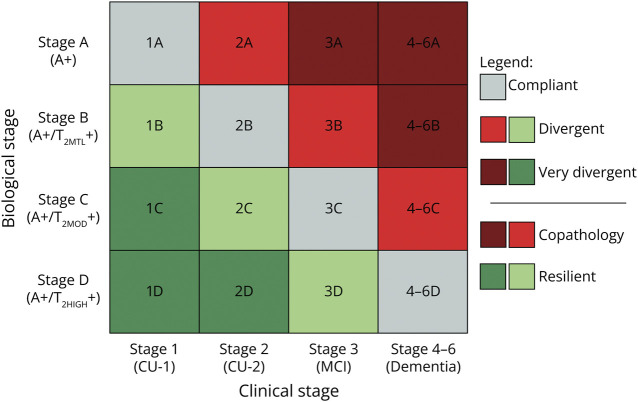
Biological and Clinical Staging Proposed by the AA-2024 Diagnostic Criteria The stages compliant with the amyloid cascade (main diagonal) are represented in gray, the divergent stages (diagonals adjacent to the main diagonal) are represented in light red and light green, and the very divergent stages (cells in the matrix's corners) are represented in dark red and dark green. The copathologic stages (off-diagonal upper matrix) are represented in light and dark red, and resilient stages (off-diagonal lower matrix) are represented in light and dark green.

A 4 × 4 clinical-biological (columns and rows, respectively) staging matrix can be developed where the diagonal represents the clinical-biological trajectory predicted by the AA-2024 operationalization of the amyloid cascade (Figure 1).^1^ Individuals above the diagonal have a more severe clinical stage than predicted based on the biological stage, suggesting the contribution of copathologies to the clinical stage. Individuals below the diagonal feature better clinical stage than predicted based on the biological stage, suggesting protection from pathology, possibly due to resilience phenomena. In populations where the amyloid cascade has full biological and clinical penetrance and there is no or negligible contribution of copathology or resilience, all patients with AD should lie on the diagonal. The most obvious example is the autosomal dominant familial AD, which is developed by variations in genes associated with β-amyloid (Aβ) production or its clearance, leading to the early and significant accumulation of amyloid plaques and consequently more predictable biological and clinical trajectories.^3^ On the contrary, in populations where the cascade has no biological and clinical penetrance, patients should distribute randomly in the 16 cells of the matrix. This scenario implies that most patients with AD exhibit copathologies or cognitive resilience because they either have additional conditions, such as cardiovascular diseases or non-related AD pathology (e.g., α-synuclein), or exhibit cognitive resilience due to being in an early clinical stage despite the presence of AD pathology. Therefore, the diagonal of the AA-2024 matrix denotes the expected trajectory of the association between clinical and pathologic stages according to the amyloid cascade hypothesis.

Understanding the number of persons identified with AD based on the AA-2024 criteria who comply or deviate from the diagonal holds important diagnostic and therapeutic implications. In a theoretical full-penetrance scenario, where the amyloid cascade is a deterministic sequence of events from disruption of amyloid metabolism to cognitive impairment, with no other pathologic pathway contributing to neurodegeneration, the detection of biomarkers of amyloid pathology in cognitively impaired persons would be sufficient to predict the future occurrence of cognitive impairment. Consequently, drugs able to completely remove amyloid deposits (e.g., anti-amyloid monoclonal antibodies) should theoretically halt the biological and cognitive progression of AD. On the contrary, in the theoretical opposite zero-penetrance scenario, biomarkers would have no predictive power and monoclonal antibodies would have minimal impact on disease progression. Therefore, in this study, we aimed to evaluate the extent to which individuals comply with or deviate from the trajectory predicted by the AA-2024 model in the Alzheimer's Disease Neuroimaging Initiative (ADNI) cohort.

## Methods

### Participants

Data from the Alzheimer's Disease Neuroimaging Initiative (ADNI)^4^ database were used for this study. The ADNI began in 2003 as a collaboration between the public and private sectors, under the leadership of Principal Investigator Michael W. Weiner, MD. The primary objective of ADNI has been to evaluate the possibility of integrating serial MRI, PET, other biological markers, and clinical and neuropsychological assessment to determine the progress from cognitively unimpaired (CU) to mild cognitive impairment (MCI) and AD dementia.

Complete information about the ADNI inclusion and exclusion criteria, as well as recruitment, is available in eAppendix 1 and Ref. 4. For this study, we have selected all CU, MCI, and dementia ADNI participants who are amyloid-positive and have had tau-PET analysis in the UC Berkeley AV1451 package, along with a neuropsychological evaluation. The amyloid positivity was determined using an amyloid-PET scan (florbetapir [18F-AV-45]).

### PET Imaging

#### Flortaucipir-PET

The ADNI UC Berkeley AV1451 Methods document published on LONI provides a comprehensive explanation of the processing of tau-PET ([18F]flortaucipir [FTP]) standardized uptake value ratio (SUVR). The regional tau-PET SUVR in the medial temporal region was extracted using the weighted average of the amygdala, entorhinal cortex, and parahippocampal gyrus. The neocortex SUVR was extracted from the weighted average of Braak 4, 5, and 6 composite regions of interest (ROIs), which included middle temporal gyrus, caudal anterior cingulate cortex, rostral anterior cingulate cortex, posterior cingulate cortex, isthmus of the cingulate cortex, insula, inferior temporal gyrus, temporal pole, superior frontal gyrus, lateral orbitofrontal cortex, medial orbitofrontal cortex, frontal pole, caudal middle frontal cortex, rostral middle frontal cortex, pars opercularis, pars orbitalis, pars triangularis, lateral occipital cortex, parietal supramarginal gyrus, parietal inferior cortex, superior temporal gyrus, parietal superior cortex, precuneus, superior temporal sulcus, and transverse temporal gyrus. As recommended by the authors of the analysis, we have performed a re-intensity normalization based on the reference region of the inferior cerebellum in medial temporal and neocortex ROIs.

#### Florbetapir-PET

The processing methods of amyloid-PET (florbetapir) can be found on LONI in the UC Berkeley AV45 Methods document. To determine the amyloid positivity in ADNI participants, we considered the cutoff of 1.11 using the whole cerebellum as a reference region, as recommended by the authors of the analysis. This cutoff corresponds to the upper bound of the 95% CI above the mean of younger healthy controls.^5^

### Clinical Staging by AA-2024: Categorization Methods

We have classified each participant into the 6 clinical stages of the AA-2024. We used the information on syndromic stage and neuropsychological profile that was collected at the closest visit to the tau-PET scan. Stages 1 and 2 included all the participants who were CU (CU-1 and CU-2; Figure 1). The CU participants in stage 2 were distinguished from those in stage 1 by the presence of subtle cognitive impairment. We used the cutoffs suggested by Shen et al.^6^ to identify subtle cognitive impairment in 4 ADNI composite scores, specifically in the domains of memory, executive functioning, language, and visuospatial. Consequently, if any CU participants had a score less than the subtle cognitive impairment cutoffs in any of the composite scores, they would be classified as stage 2. Stage 3 included patients with MCI, and stages 4–6 included patients with dementia.

### Biological Staging by AA-2024: Methods for Determining Cutoff Points

The biological staging proposed by AA-2024 reflects a sequence from initial to advanced stages of AD relying on the magnitude and topography of tau deposition in amyloid-positive individuals. Specifically, stage A includes participants who are positive for amyloid-PET (A+). Stage B comprises individuals who are A+ and positive for tau-PET in the medial temporal region (A+/T_2MTL_+). Stage C encompasses participants who are positive for both aforementioned criteria and exhibit moderate neocortical tau-PET uptake (A+/T_2MOD_+). Finally, Stage D comprises participants with high neocortical tau-PET uptake (A+/T_2HIGH_+).

To find cutoff points for SUVR positivity in medial temporal and neocortex regions from tau-PET, we used 5 different methods suggested by Jack et al.^7^ The cutoff points were determined using reliable worsening based on longitudinal change (RW), specificity based on young clinically normal (Spec), sensitivity based on cognitively impaired amyloid-positive (Sens), accuracy based on young clinically normal vs cognitively impaired amyloid-positive (Acc-Young), and accuracy based on age-matched clinically normal vs cognitively impaired amyloid-positive (Acc-Matched). Furthermore, we calculated an additional cutoff for the neocortex ROI, based on the median of the group positive for neocortical tau-PET uptake (A+/T_2MOD+_ and A+/T_2HIGH+_), to distinguish between moderate and high SUVRs.

The cutoff point by the RW method was calculated using the ADNI participants with at least 2 tau-PET scans. Using linear regression, we initially calculated the annual rate of change of SUVR in medial temporal and neocortex ROIs for each participant based on their sequence of PET scans. With these data, we plotted a nonparametric scatter plot using the Loess method with the mean rate of change on the y-axis in relation to the baseline SUVR on the x-axis. Then, using a 50% prediction interval, we identified the minimum point of our Loess curve and calculated its upper bond. Finally, we projected the upper bond rightward until it intersected with the Loess curve, where we found our RW cutoff point.

The Spec method included the 30% of younger ADNI participants who were CU and amyloid-negative. The cutoff was set at the 95th percentile of the medial temporal and neocortex SUVR distribution. On the contrary, the Sens method comprised participants with MCI or dementia (cognitively impaired [CI]) who were older than 60 years and tested positive for amyloid. The cutoff value was determined based on the 10th percentile of the distribution of SUVR in both ROIs.^7^

The Acc-Young method aims to distinguish between the 2 groups of participants included in Spec and Sens methods. Specifically, this method focuses on differentiation between younger CU individuals who are amyloid-negative and older individuals with MCI or dementia who are amyloid-positive. For that, the cutoff point is determined by calculating the largest difference between the smoothed cumulative distribution functions (CDFs) of the 2 groups, with the goal of maximizing accuracy. Likewise, the Acc-Matched followed a similar analysis but discriminated between the older individuals with MCI or dementia who are amyloid-positive and a control group comprising amyloid-negative CU participants matched for age and sex to the CI group. To ensure a balanced comparison between groups, we used the *matchit()* function from the MatchIt package^8^ from R to perform nearest neighbor matching in all ADNI participants with at least one tau-PET scan. More precisely, we used nearest neighbor matching to match participants based on age and exact matching to match them based on sex. Then, the cutoff was determined using the same approach as the Acc-Young method, which involved calculating the largest difference between the smoothed CDFs of both groups.

The demographics and clinical and imaging characteristics of each sample used in each method are presented in eTable 1.

### Statistical Analysis

To evaluate our main objective, the participants were categorized according to the AA-2024 biological and clinical stage. Then, we classified the individuals based on their alignment with the expected trajectory of the amyloid cascade, according to 3 categories: (1) compliant with the expected trajectory of the amyloid cascade (represented by the matrix's main diagonal), (2) divergent (represented by the 2 diagonals adjacent to the main diagonal), and (3) very divergent (represented by the cells in the matrix's corners) (Figure 1). In addition, we classified “copathologic” individuals as those with clinical stage worse than expected based on their biological stage (i.e., off-diagonal upper matrix) and “resilient” individuals as those with clinical stage better than what would be expected based on their biological stage (i.e., off-diagonal lower matrix) (Figure 1). The proportion of participants who fall into each category was calculated using the 5 methods for determining tau-PET cutoffs. We conducted a χ^2^ goodness-of-fit test to compare the observed distributions of clinical and biological stages with 2 hypothetical distributions. The first hypothetical distribution assumes an equal probability for each stage, representing a scenario where each stage is equally likely (i.e., zero-penetrance scenario). The second hypothetical distribution represents a high penetrance of the amyloid cascade, where 50% of the individuals fall into the main diagonal cells (eFigure 1). Finally, to analyze and evaluate the distribution of participants across different clinical and biological stages using the 5 approaches, we conducted a Cochran Q test and post hoc pairwise McNemar tests. The results were corrected using the Holm method. All the analyses were performed in R (version 4.2.3).^9^

### Standard Protocol Approvals, Registrations, and Patient Consents

Written informed consent was obtained from all participants in the ADNI study, in accordance with the requirements of local institutional review boards (IRBs). The most current information is available at adni-info.org.

### Data Availability

The Alzheimer's Disease Neuroimaging Initiative (ADNI) database can be accessible at ida.loni.usc.edu on request.

## Results

### Participants

We identified 256 amyloid-positive individuals from the ADNI database with a tau-PET FTP. Our sample was composed of 111 CU participants, 90 participants with MCI, and 55 participants with dementia. Table 1 provides the demographics and clinical and imaging characteristics of each clinical stage proposed by AA-2024.

**Table 1:** Characteristics of the ADNI Participants by Clinical Stage

	Clinical stage 1 CU-1 (n = 60)		Clinical stage 2 CU-2 (n = 51)		Clinical stage 3 MCI (n = 90)		Clinical stages 4–6 Dementia (n = 55)		*p* Value
Demographics/clinical characteristics									
Age, y	70.4 ± 6.3^b^	60	74.6 ± 6.7^b^	51	72.5 ± 6.6	89	73.9 ± 7.9^a^	55	0.006
Sex, female	44 (73%)^a^	60	23 (45%)	51	40 (44%)^b^	90	23 (41%)^b^	55	0.001
Education, y	16.8 ± 2.2^a^	60	16.5 ± 2.6	51	16.1 ± 2.7	90	15.6 ± 2.4^b^	55	0.06
ADAS-13 score	6.6 ± 3.5^d^	60	11.7 ± 5.3^c^	50	17.5 ± 6.5^b^	88	31.3 ± 9.3^a^	53	<0.001
MMSE score	29.3 ± 0.9^a^	60	28.3 ± 1.7^a^	51	27.1 ± 2.2^b^	90	21.7 ± 4.3^c^	55	<0.001
Memory composite	1.4 ± 0.62^a^	60	0.61 ± 0.52^b^	51	0.06 ± 0.61^c^	90	−0.91 ± 0.66^d^	55	<0.001
EF composite	1.11 ± 0.67^a^	59	0.53 ± 0.62^b^	50	0.15 ± 0.89^c^	89	−0.95 ± 0.92^d^	54	<0.001
Language composite	1.18 ± 0.65^a^	60	0.36 ± 0.57^b^	51	0.17 ± 0.84^b^	90	−0.77 ± 0.89^c^	55	<0.001
Visuospatial composite	0.39 ± 0.51^a^	60	−0.08 ± 0.85^b^	51	−0.03 ± 0.82^b^	90	−0.63 ± 1.17^c^	55	<0.001
APOE4 carriers	32 (53%)	60	22 (42%)	51	55 (61%)	90	32 (58%)	55	0.21
Imaging									
Amyloid-PET SUVR	1.28 ± 0.15^b^	60	1.32 ± 0.19^b^	49	1.42 ± 0.2^a^	83	1.48 ± 0.19^a^	46	<0.001
Medial temporal tau SUVR	1.19 ± 0.14^c^	60	1.22 ± 0.13^c^	51	1.41 ± 0.26^b^	90	1.54 ± 0.32^a^	55	<0.001
Neocortical tau SUVR	1.11 ± 0.08^c^	60	1.13 ± 0.08^c^	51	1.22 ± 0.19^b^	90	1.38 ± 0.39^a^	55	<0.001
Hippocampal volume	7,491 ± 736^a^	46	7,055 ± 863^a^	39	6,722 ± 966^b^	70	5,802 ± 962^c^	36	<0.001
FDG-PET SUVR	1.36 ± 0.06^a^	3	1.26 ± 0.07	2	1.18 ± 0.14^b^	48	1.05 ± 0.13^c^	30	<0.001

Abbreviations: ADAS-13 = Alzheimer's Disease Assessment Scale 13; APOE4 = apolipoprotein E ε4; MMSE = Mini-Mental State Examination; SUVR = standardized uptake value ratio.

Mean ± SD for continuous variables and frequency (%) for categorical variables. The *p* value corresponds to the one-way ANOVA for continuous variables and χ^2^ test for categorical variables. The post hoc test of significance corrected for multiple comparisons is represented by superscript letters indicating a>b>c>d. CU-1: cognitively unimpaired. CU-2: cognitively unimpaired with subtle impairment.^6^ MCI: mild cognitive impairment. Dementia: mild, moderate, and severe corresponding to clinical stages 4–6. Amyloid-PET SUVR: cortical gray matter/cerebellum. FDG-PET SUVR: meta ROI.

### Compliances and Divergences With the Expected Trajectory of the Amyloid Cascade

**Figure 2:**
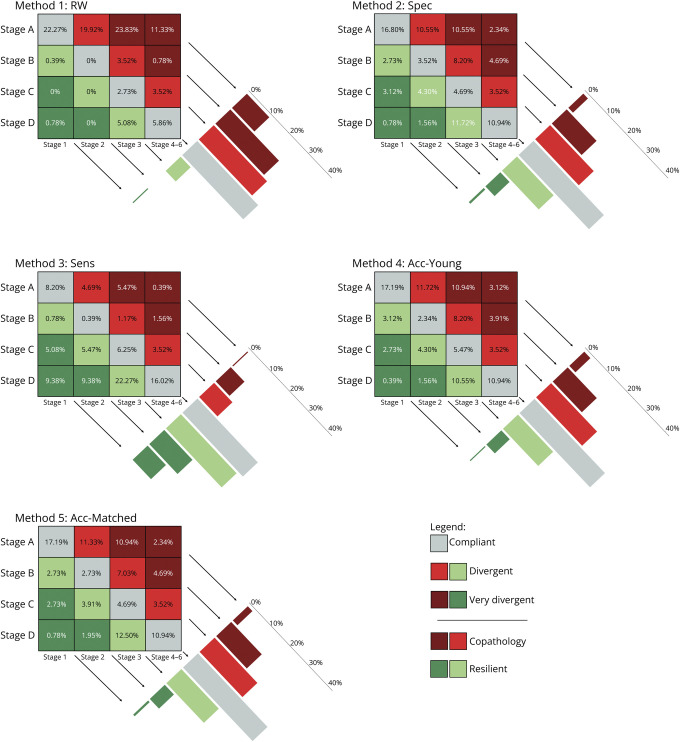
Distribution of the ADNI Participants Along the Biological and Clinical Staging Estimated Based on the 5 Cutoff Methods The proportion of participants compliant with the amyloid cascade was between 31% and 36% while the proportion of copathologic individuals ranged between 17% and 63% and resilient individuals ranged between 6% and 52%. ADNI = Alzheimer’s Disease Neuroimaging Initiative.

The proportion of participants complying with the amyloid cascade ranged from 30.9% to 35.9% across the 5 methods used for determining tau-PET cutoff points (Figure 2). The proportion of participants who diverged from the expected trajectory and were located in the 2 adjacent diagonals had a range of 32.3%–41.4%. In addition, the proportion of participants who diverged the most from the expected trajectory and were located in the corners of the matrix ranged from 22.4% to 36.6% (Table 2; Figure 2).

**Table 2:** Cutoff Points and Proportion of Patients (%) With Underlying 95% CI in Each Category Regarding Their Biological and Clinical Staging Based on Each Method of Defining Cutoff Points

Methods	Reliable worsening	Specificity	Sensitivity	Accuracy-young	Accuracy-matched
Cutoffs					
Medial temporal ROI	1.51	1.23	1.13	1.24	1.23
Neocortex ROI (moderate SUVR)	1.25	1.15	1.04	1.16	1.15
Neocortex ROI (high SUVR)	1.36	1.24	1.12	1.25	1.23
Proportion of patients (%)					
Compliant	31.1 (25.6–36.9)	35.9 (30–42.2)	30.9 (25.3–36.9)	35.9 (30–42.2)	35.6 (29.7–41.8)
Divergent	32.3 (26.7–38.5)	41 (34.9–47.3)	37.9 (31.9–44.1)	41.4 (35.3–47.7)	41 (34.9–47.3)
Very divergent	36.6 (30.8–42.9)	23.1 (18–28.7)	31.3 (25.6–37.3)	22.7 (17.7–28.3)	22.4 (17.3–27.9)
Copathology	63.4 (57–69.2)	39.8 (33.8–46.1)	16.8 (12.4–21.9)	41.4 (35.3–47.7)	39.8 (33.8–46.1)
Resilient	5.5 (3–9)	24.2 (19.1–29.9)	52.3 (46–58.6)	22.7 (17.7–28.3)	24.6 (19.5–30.4)

Abbreviation: ROI = region of interest.

Reliable worsening: based on longitudinal change. Specificity: based on young clinical normal. Sensitivity: based on cognitively impaired amyloid-positive. Accuracy-Young: based on young clinically normal vs cognitively impaired amyloid-positive. Accuracy-Matched: based on age-matched clinically normal vs cognitively impaired amyloid-positive.

**Figure 3:**
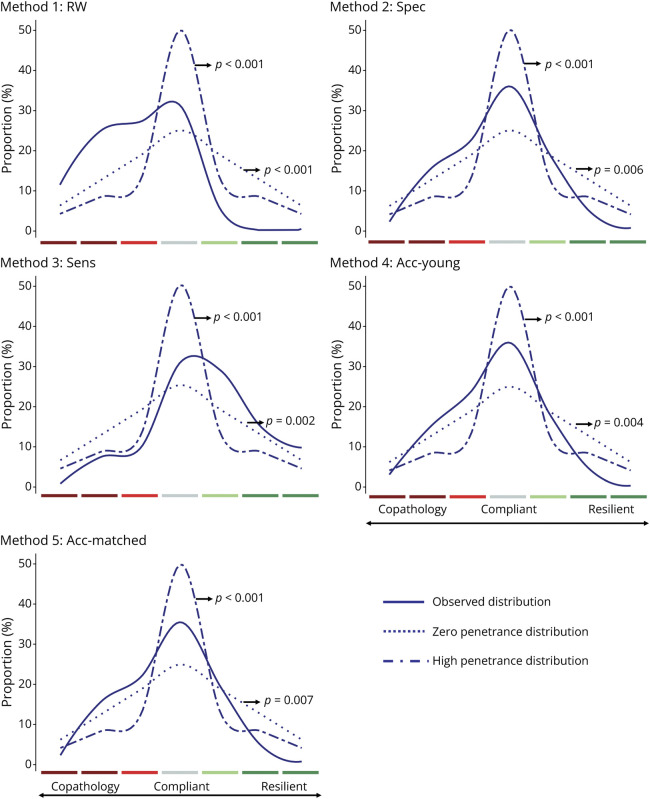
Smoothed Curves Representing 3 Distributions—Observed (Solid Line), Zero Penetrance (Dotted Line), and High Penetrance (Dashed Line)—Across the 5 Cutoff Methods The *p* values refer to the significance observed in the χ^2^ goodness-of-fit test between the observed distribution and the hypothetical high/zero-penetrance distributions.

The χ^2^ goodness-of-fit test revealed significant differences between the 5 method distributions and the zero-penetrance distribution (RW: χ^2^(6) = 49.5, *p* < 0.001; Spec: χ^2^(6) = 18.2, *p* = 0.006; Sens: χ^2^(6) = 20.9, *p* = 0.002; Acc-Young: χ^2^(6) = 18.9, *p* = 0.004; Acc-Matched: χ^2^(6) = 17.9, *p* = 0.007), because of a higher proportion of individuals falling into the expected pathway (main diagonal) and different tail distributions for the 5 methods compared with the equal probability distribution (Figure 3). Likewise, we also observed significant differences between the observed distributions using the 5 methods and the high-penetrance distribution (RW: χ^2^(6) = 85.6, *p* < 0.001; Spec: χ^2^(6) = 25.6, *p* < 0.001; Sens: χ^2^(6) = 43.3, *p* < 0.001; Acc-Young: χ^2^(6) = 26.6, *p* < 0.001; Acc-Matched: χ^2^(6) = 26.2, *p* < 0.001). The high-penetrance distribution revealed a higher proportion of individuals along the amyloid cascade diagonal in comparison with the 5 distributions, as well as different resilient and copathology distributions (Figure 3).

Regarding the differences among the 5 methods, the Cochran Q test revealed no statistically significant differences in the proportion of participants in the expected trajectory or in the divergent pathway (*p* < 0.1). However, the Cochran Q test revealed significant differences among the 5 methods in the proportion of very divergent participants (*Q*(4) = 40.3, *p* < 0.001). The post hoc pairwise comparison showed that RW had a significantly higher proportion of very divergent individuals in comparison with Spec (*p* < 0.001), Acc-Young (*p* < 0.001), and Acc-Matched (*p* < 0.001) methods.

### Differences Between Copathologic and Resilient Individuals

The categorization of copathologic and resilient individuals showed a large variability depending on the method chosen to find the cutoff, namely ranging from 16.8% to 63.4% and 5.5% to 52.3%, respectively. The Cochran Q test revealed differences among the 5 methods in the proportion of copathologic (*Q*(4) = 293, *p* < 0.001) and resilient (*Q*(4) = 301, *p* < 0.001) individuals. The Sens method revealed significantly fewer copathologic individuals compared with the other 4 methods (*p* < 0.001). The Spec and Acc-Matched methods also showed significantly fewer copathologic individuals than RW and Acc-Young methods (*p* < 0.001). Acc-Young had significantly fewer copathologic individuals than RW (*p* < 0.001). A similar pattern emerged in the post hoc comparisons of methods evaluating the proportion of resilient individuals. Specifically, the Sens method identified significantly more resilient individuals compared with the other 4 methods (*p* < 0.001). The Spec method showed a significantly higher number of resilient individuals than RW and Acc-Matched methods (*p* < 0.001). The Acc-Young and Acc-Matched methods also identified significantly more resilient individuals than RW (*p* < 0.001).

### Cutoff Points in Tau-PET

The cutoff point using the RW method was set at 1.51 for the medial temporal region (A+/T_2MTL+_) and 1.25 and 1.36 for moderate (A+/T_2MOD+_) and high (A+/T_2HIGH+_) neocortical uptake, respectively. The Spec and Acc-Matched methods revealed similar cutoff points: 1.23 in the medial temporal region, 1.15 for moderate neocortical uptake, and 1.24 and 1.23 for high neocortical uptake, respectively. The Sens method revealed a cutoff point of 1.13 for the medial temporal region and 1.04 and 1.12 for the moderate and high uptake in the neocortex, respectively. Last, the Acc-Young method found a cutoff point of 1.24 in the medial temporal ROI and 1.16 and 1.25 for moderate and high neocortical tau uptake. In general, the RW method was the most conservative approach for establishing cutoff points while the Sens method was shown to be the most lenient approach (Table 2). All scatter and CDF plots used to estimate the cutoffs are presented in eFigures 2–6.

## Discussion

In this study, we showed that the number of individuals in the expected trajectory proposed by the AA-2024 never exceeded 36% regardless of the method used to determine the biological staging. If we consider that the expected trajectory is a representation of the amyloid cascade pathway, we observed that only approximately one-third of the individuals follow with this direction while the proportion of individuals diverging from the expected trajectory ranged between 64% and 69%, regardless of the biological staging method. Moreover, our results showed that the number of individuals compliant with the amyloid cascade pathway is slightly larger than anticipated when assuming that each stage is equally probable (zero-penetrance scenario—25% of compliance); however, it is still lower than what would be expected in a situation with a high penetrance of the amyloid cascade (high-penetrance scenario—50% of compliance).

Our findings suggest that the amyloid cascade can be modulated by nonamyloid cascade factors and the expected trajectory does not manifest clinically as expected in most of the cases. Individuals who are *APOE* ε4 homozygotes exhibit a higher penetrance of the amyloid cascade demonstrated by the different amyloid and tau distributions when compared with noncarriers.^3,10^ By using the clinical-biological trajectory suggested by AA-2024, we can infer that the compliance with the amyloid cascade can be influenced by *APOE* ε4 genotype. In addition, several environmental factors, such as education, social isolation, and air pollution, have been demonstrated to contribute to the pathophysiology and underlying symptoms of dementia. For example, individuals with higher levels of education who have MCI presented a certain degree of protection against tauopathy, as seen by a decreased tau burden, compared with individuals with lower education.^11,12^ Another study has demonstrated that socially isolated individuals reveal different patterns of tau deposition, namely with a greater accumulation in the right entorhinal region taking into consideration age, sex, and *APOE* ε4.^13^ Air pollution exposure in CU individuals has been associated with increased levels of Aβ in the brain and neurofilament light chain in CSF.^14^ Of interest, a similar effect has already been observed in younger individuals who have been exposed to air pollution.^15^ These previous findings suggest that environmental factors may play a role in the progression of AD pathology and might affect the relationship between the biological and the clinical stage. Last, there are clinical factors associated with cardiovascular health that play a significant role in the pathophysiology and progression of AD. Elevated levels of tau in CSF were found to be associated with hypertension, lack of obesity, and a high Framingham Risk Score, although no effect was observed in the Aβ accumulation.^16^ Likewise, similar results were found in a memory clinic cohort, where the presence of different vascular risk factors led to different AD pathology profiles. For instance, individuals with carotid artery stenosis showed higher levels of CSF Aβ42 and total tau, whereas those with diabetes and alcohol abuse presented greater medial temporal atrophy.^17^ Last, the existence of cerebrovascular pathology also has been demonstrated to alter the AD clinical trajectory. This is because the presence of vascular pathology in the brain was also associated with increased accumulation of tau in amyloid-positive individuals.^18^

Overall, these findings are congruent with the hypothesis that the expected trajectory of the amyloid cascade might be influenced by genetic, environmental, and clinical factors, leading to divergences along the AD continuum.^19^ Similarly, the number of individuals in the expected trajectory may be influenced by the existence of individuals with copathologies and resilience to the pathology. In fact, in our study, we observed statistically significant differences in the number of individuals classified as copathologic and resilient among the cutoff approaches. The proportion of individuals classified as resilient and copathologic varies considerably between the 5 methods, with some ways increasing the number of copathologic individuals (ranging between 16.8% and 63.4%) and others increasing the number of resilient individuals (ranging between 5.5% and 52.3%) (Figure 2). Patients with AD commonly experience multiple copathologies, with hypertension, osteoarthritis, depression, diabetes mellitus, and cerebrovascular disease being the most prevalent.^20^ The presence of these copathologies in AD is also observed in other neurodegenerative diseases, such as Lewy body disease^21^ and Parkinson disease.^22^ For instance, in the ADNI cohort, the prevalence of α-synuclein among individuals with AD was 22%, worsening with disease progression and age.^23^ The synuclein copathology can exacerbate cognitive decline in neurodegenerative diseases by interacting synergistically with Aβ and tau pathology, which affects the clinical manifestation of AD symptoms.^24^ Similarly, a recent study has demonstrated that only 41% of clinically diagnosed cases of AD are attributable to classical AD pathology while the remaining cases were attributed to other pathologies such as macroscopic infarcts, Lewy bodies, hippocampal sclerosis, and transactive cerebral amyloid angiopathy, among others.^25^ Another study found a similar percentage of individuals with pure AD pathology with early onset, namely 44%, which decreased to 20% if we consider individuals older than 70 years.^26^ Of interest, the proportion of individuals that comply with the expected trajectory in our study (31%–36%) is comparable with the previous prevalence of individuals with pure AD pathology.^25^ On the contrary, the resilience to the pathology shown by an individual also influences the projected trajectory of the disease. Current studies have shown that the percentage of patients with AD pathology who demonstrate cognitive resilience ranges between 9% and 30%,^27-29^ which is consistent with our own findings to some extent.

Our results suggest that the presence of biomarkers indicating AD does not explain the clinical symptomatology in approximately two-thirds of the cases. Specifically, we observed that among most of the participants who are CU (stages 1 and 2), the distribution and magnitude of amyloid and tau are not explaining their lack of clinical symptomatology. Likewise, in CI individuals, we observe the same pattern but in the opposite direction, where amyloid and tau alone are not enough to explain their symptomatology. These cases can be better understood by considering copathologies because the cognitive impairment observed is not consistent with the pathology of AD.

Translating these findings to the treatment field, our results suggest that the efficacy of disease-modifying drugs targeting the amyloid cascade may vary widely among patients because the clinical status of many individuals with AD pathology seems to be only marginally influenced by this trajectory. In fact, the inclusion of individuals with a copathologic or resilient profile in clinical trials can reduce the statistical power due to the population heterogeneity. This is in line with one of the main criticisms of the amyloid cascade hypothesis, which is the inconsistency of results in many clinical trials targeting Aβ. Some of these trials have not been able to demonstrate clinical gains,^30^ until the recent exceptions of lecanemab and donanemab.^31,32^ Nonetheless, assuming that anti-Aβ drugs achieve their best theoretical efficacy, we would anticipate more consistent results if all individuals in the AD continuum revealed a high penetrance of the cascade and consequently followed a deterministic sequence consistent with the amyloid cascade hypothesis.

Last, we established tau-PET cutoff points for medial temporal and neocortex ROIs to allow the categorization of the biological staging proposed by AA-2024, using 5 distinct methods (Table 2). We have observed similar cutoff points using Spec and Acc-Matched methods and more conservative cutoffs when applying the RW method. The Spec, Sens, and Acc-Young methods have already shown similar cutoffs for tau-PET, whereas the Acc-Matched method also revealed a more conservative cutoff.^7^ This highlights the importance of the accurate determination of tau-PET cutoffs for accurately categorizing biological staging because there has been significant variability in cutoff points reported in the literature.^33^ Hence, in this study, we proposed different cutoffs for the ADNI tau-PET files by using the recommended ROIs.

Our study used the ADNI database, which consists of a research sample with similar inclusion and exclusion criteria of AD clinical trials, rather than a memory clinic cohort.^34^ As a result, the ADNI cohort comprises individuals who are more educated and have a higher socioeconomic status, while also significantly underrepresenting ethnocultural groups such as Black and Latino populations.^35^ Furthermore, at the pathologic level, the ADNI cohort excludes some participants with vascular copathology (i.e., Hachinski Ischemic Score >4^36^). Hence, it is reasonable to speculate that if a more representative group of individuals is included in the study, the proportion of individuals in the expected trajectory may be modulated because of the higher variability in other factors such as genetic, environmental, and clinical characteristics. The impact of these factors, such as copathologies, *APOE* ε4 genotype, and ethnocultural groups, should be tested in future studies with larger sample sizes that allow subgroup analysis.

In addition, AA-2024 has identified several caveats to the biological staging that should also be acknowledged in our work. For instance, the tauopathy in medial temporal regions does not consistently occur before neocortical tauopathy,^37^ despite it being the most prevalent pattern.^38^ Moreover, the AA-2024 does not provide a set of anatomical ROIs for identifying the medial temporal or the neocortex regions. In fact, it is suggested that some brain areas, such as the hippocampus, entorhinal cortex, and amygdala, could potentially be included in the medial temporal ROI, or the inferior and medial parietal lobes could be included in the neocortex ROI. Consequently, we followed the recommendations of AA-2024 and implemented the regions proposed by AA-2024 for the medial temporal region, except the hippocampus because of the contamination by off-target binding in the choroid plexus. In the neocortex ROI, we have selected a comprehensive neocortex ROI using Braak 4, 5, and 6 from Freesurfer-defined composite ROIs.

Another limitation pointed by AA-2024 is how the distinction between moderate (stage C) and high (stage D) neocortical tau uptake should be evaluated. In our study, we chose to follow the 5 methods proposed by Jack at al.^7^ to evaluate the presence of tau in both regions, while to evaluate the distinction between moderate and high uptake in the neocortex ROI, we have used the median in the A+/T_2MOD+_ and A+/T_2HIGH+_ individuals. Nevertheless, the estimation of this cutoff point should be further tested using different methods in future research because it has not been adequately investigated in previous studies.

This study demonstrated that only approximately one-third of the amyloid-positive individuals from ADNI population comply with the expected trajectory proposed by AA-2024. This was seen irrespective of the chosen tau-PET threshold. Moreover, we showed that most of the participants deviate from the anticipated AD course, likely because of copathologies or cognitive resilience, suggesting a low penetrance of the amyloid cascade. Overall, our findings emphasize the high variability that characterizes AD, which could be attributed to genetic, environmental, and clinical factors. In addition, we have also included 5 cutoff points for both the medial temporal and neocortex ROI, which can be used to categorize the AA-2024 biological staging in ADNI.

## Appendix Coinvestigators

**Table:**

Alzheimer's Disease Neuroimaging Initiative coinvestigators are listed in the Supplementary Material at Neurology.org/N

## Glossary

AD: Alzheimer disease
ADNI: Alzheimer's Disease Neuroimaging Initiative
Aβ: β-amyloid
CDF: cumulative distribution function
CU: cognitively unimpaired
MCI: mild cognitive impairment
ROI: region of interest
SUVR: standardized uptake value ratio
